# The value of lung ultrasound in COVID-19 pneumonia, verified by high resolution computed tomography assessed by artificial intelligence

**DOI:** 10.1186/s12879-023-08173-4

**Published:** 2023-03-31

**Authors:** Robert Chrzan, Kamil Polok, Jakub Antczak, Andżelika Siwiec-Koźlik, Wojciech Jagiełło, Tadeusz Popiela

**Affiliations:** 1 Department of Radiology, Jagiellonian University Medical College, Kopernika 19, 31-501 Krakow, Poland; 2 Department of Intensive Care and Perioperative Medicine, Jagiellonian University Medical College, Krakow, Poland; 3 Department of Neurology, Jagiellonian University Medical College, Krakow, Poland; 4 Department of Rheumatology and Immunology, Jagiellonian University Medical College, Krakow, Poland; 5 Second Department of Internal Medicine, Jagiellonian University Medical College, Krakow, Poland

**Keywords:** Artificial intelligence, LUS, HRCT, COVID-19

## Abstract

**Background:**

Lung ultrasound (LUS) is an increasingly popular imaging method in clinical practice. It became particularly important during the COVID-19 pandemic due to its mobility and ease of use compared to high-resolution computed tomography (HRCT). The objective of this study was to assess the value of LUS in quantifying the degree of lung involvement and in discrimination of lesion types in the course of COVID-19 pneumonia as compared to HRCT analyzed by the artificial intelligence (AI).

**Methods:**

This was a prospective observational study including adult patients hospitalized due to COVID-19 in whom initial HRCT and LUS were performed with an interval < 72 h. HRCT assessment was performed automatically by AI. We evaluated the correlations between the inflammation volume assessed both in LUS and HRCT, between LUS results and the HRCT structure of inflammation, and between LUS and the laboratory markers of inflammation. Additionally we compared the LUS results in subgroups depending on the respiratory failure throughout the hospitalization.

**Results:**

Study group comprised 65 patients, median 63 years old. For both lungs, the median LUS score was 19 (IQR—interquartile range 11–24) and the median CT score was 22 (IQR 16–26). Strong correlations were found between LUS and CT scores (for both lungs *r* = 0.75), and between LUS score and percentage inflammation volume (PIV) (*r* = 0.69). The correlations remained significant, if weakened, for individual lung lobes. The correlations between LUS score and the value of the percentage consolidation volume (PCV) divided by percentage ground glass volume (PGV), were weak or not significant. We found significant correlation between LUS score and C-reactive protein (*r* = 0.55), and between LUS score and interleukin 6 (*r* = 0.39). LUS score was significantly higher in subgroups with more severe respiratory failure.

**Conclusions:**

LUS can be regarded as an accurate method to evaluate the extent of COVID-19 pneumonia and as a promising tool to estimate its clinical severity. Evaluation of LUS in the assessment of the structure of inflammation, requires further studies in the course of the disease.

**Trial registration:**

The study has been preregistered 13 Aug 2020 on clinicaltrials.gov with the number NCT04513210.

## Background

The lung ultrasound (LUS) is an increasingly used diagnostic option in pulmonary medicine [1]. It gained special attention during COVID-19 pandemic, when the need for assessment and monitoring of pneumonia increased dramatically. Contrary to the high-resolution computed tomography (HRCT) and X-ray of the chest, LUS is relatively inexpensive, which allows to provide higher number of devices to healthcare facilities. For monitoring of disease progression LUS can be done repetitively without exposing the patient to ionizing radiation. LUS is also associated with significantly lower risk of COVID-19 and other infectious transmission as particular devices may be dedicated exclusively to infected patients and the sterilization is simple [2]. Moreover as the ultrasound devices are easily portable, LUS can be performed in critically ill and unstable patient [3].

According to initial reports, changes in LUS typical for COVID-19 pneumonia include the presence of vertical, hyperechogenic B-lines, which are the expression of interstitial changes. B-lines obscure the physiologic, horizontal A-lines, which are reverberation artifact of the pleura signal. In more severely affected lung areas B-lines may coalesce to hyperechogenic areas occupying significant part of the field. Another finding, associated with chronic stage of pneumonia is the consolidation, reflected by subpleural hypoechoic areas with signal resembling that of parenchymal organs i.e. liver [1]. Inspection of the lungs with LUS is often systematized by dividing the chest into segments and scoring every segment with respect to severity of visualized changes.

The most common HRCT findings in COVID-19 pneumonia include ground glass opacities, vascular redistribution, followed by air-bronchogram, consolidations and crazy paving pattern [4]. Typically both lungs are involved, with peripheral distribution of lesions, involvement of more than one lobe and predilection to lower lobes.

At the beginning of the COVID-19 pandemic, it was suggested to use chest HRCT for the verification of positive cases, particularly in hospitals with a huge number of new patients and insufficient access to polymerase chain reaction (PCR) tests [5].

However, it turned out, that just the same radiological symptoms may occur in pneumonia of a different etiology, particularly atypical, which limited the specificity of the HRCT.

For this reason the radiological societies, like British Thoracic Imaging Society and American College of Radiology do not recommend HRCT as a COVID-19 screening tool, nor as a first-line test, and PCR from the pharyngeal or nasopharyngeal swab is still the only reliable method of verification. However, the guidelines suggest that HRCT can be used in complicated COVID-19 cases [6, 7].

Artificial intelligence (AI) software performing automatic assessment of X-ray or computed tomography (CT) images can be a valuable tool in daily practice. [8, 9] In particular, AI can rapidly assess a huge number of images, which is very important in centers with limited number of trained staff [10].

The specificity of the automatic determining COVID-19 as an etiological factor in pneumonia is still limited [10–12]. However, AI assessment of HRCT scans can objectively assess the volume of infiltration in patients with COVID-19 confirmed by PCR, in the course of treatment, which is very important for optimization of therapy and early prediction of severe course with possible need of ventilation [13–15]. Despite vast availability and other advantages of LUS, the data concerning its ability to monitor and prognose the COVID-19 pneumonia are still very scarce.

The main objective of this prospective observational study was therefore to assess the value of LUS in quantifying the degree of lung involvement and in discrimination of lesion types in the course of COVID-19 pneumonia as compared to chest HRCT analyzed by AI software.

Furthermore, we investigated the value of LUS in assessment of disease severity, regarding laboratory markers of inflammation and respiratory failure.

## Methods

### Study setting and design

This is a substudy of the CRACoV-HHS (CRAcow in CoVid pandemic — Home, Hospital and Staff) project, a prospective observational study enrolling consecutive adult patients hospitalized due to COVID-19 infection. Study participants were recruited from July 2020 to May 2021 in the University Hospital in Kraków, Poland. The study protocol complied with Helsinki Declaration and was approved by the Jagiellonian University Ethical Board (approval number 1072.6120.333.2020, December 7, 2020). The design and conduct of the CRACoV-HHS project were described in detail previously [16].

### Study population

The general CRACoV-HHS study sample included patients aged ≥ 18 years who were hospitalized due to COVID-19 confirmed with reverse-transcription polymerase chain reaction (RT-PCR) test from nasopharyngeal swabs. In every patient in this substudy initial LUS and chest HRCT were performed, with an interval between the two examination < 72 h. The exclusion criteria were: previously diagnosed interstitial lung disease, chronic respiratory failure, acute respiratory failure defined as hemoglobin oxygen saturation (SpO_2_) on room air < 94% at admission, clinical signs of severe airway obstruction, pulmonary embolism within prior 3 months, pneumothorax, pulmonary congestion, pleural fluid > 500 ml during initial examination, hemodynamic instability, and chest deformation preventing reliable LUS examination.

The CRACoV-HHS project aimed to optimize patient care and assumed the use of LUS as a tool for the initial assessment and monitoring the course of the disease, including the prediction of clinical deterioration and the need for mechanical ventilation. For this reason, this study did not include patients with respiratory insufficiency at the admission.

### Lung ultrasound examination

**Fig. 1:**
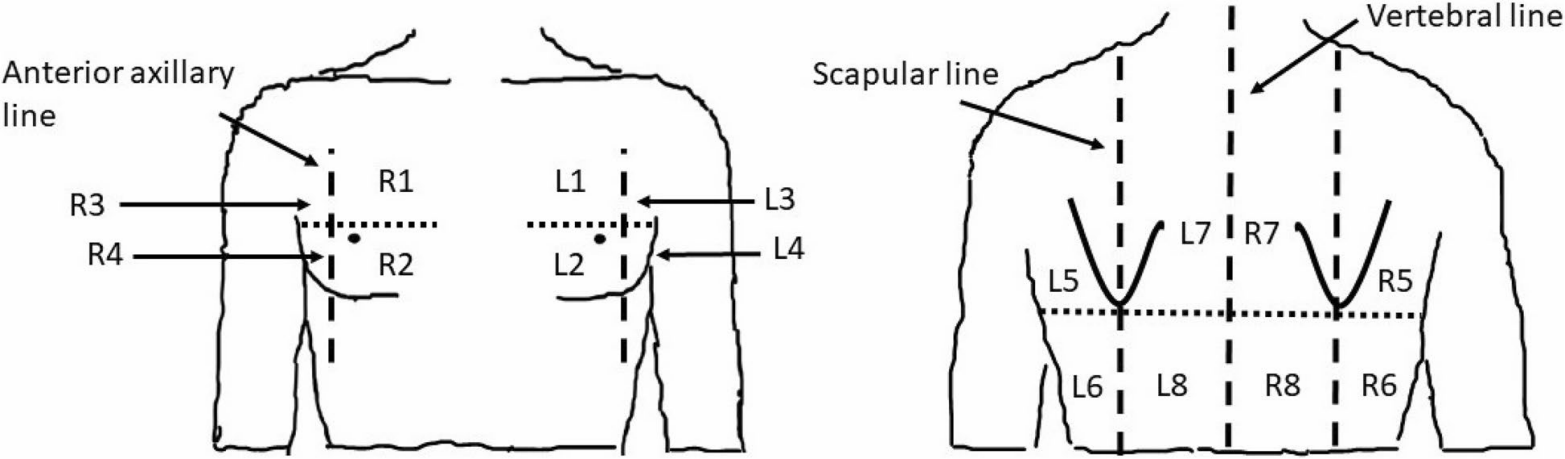
The segments of chest area used during LUS

The protocol of LUS examination was adopted from the method described by Bouhemad et al. [17] with modification introduced by European Association of Cardiovascular Imaging. The chest area was divided into eight segments on the left and eight segments on the right side (Fig. 1).

Every segment was then rated according to scale ranging from 0 to 3, where 0 meant normal lung echogenicity, 1 moderate loss of aeration, reflected by the presence of at least three „B” lines or coalescent lines covering less than 50% of the screen, 2 „B” lines covering more than 50% of the screen, and 3 the signs of consolidation. The scores for both lungs, right and left lung were calculated as the sums of points from the corresponding areas. Additionally, in order to estimate the involvement of individual lobes, we assigned the above chest area segments used during LUS to the approximate anatomical locations of every lobe (Table 1).

**Table 1:** The assignment of the chest area segments used during LUS to the approximate anatomical locations of every lobe

LUS chest area segments	Anatomical lobe
R1,R3,R5,R7	Right upper lobe
R2	Right middle lobe
R4,R6,R8	Right lower lobe
L1,L2,L3,L5,L7	Left upper lobe
L4,L6,L8	Left lower lobe

The scores for every lobe were then calculated as the sums of points from the corresponding areas.

The doctors performing LUS did not know the results of the chest HRCT examination.

### High-resolution computed tomography and artificial intelligence assessment

Chest HRCT scanning was performed by multirow (64 or 80) helical scanners, using the following parameters: tube current–time product 100 – 350 mAs, voltage 120 kV, slice thickness 0.625 – 1.25 mm.

**Fig. 2:**
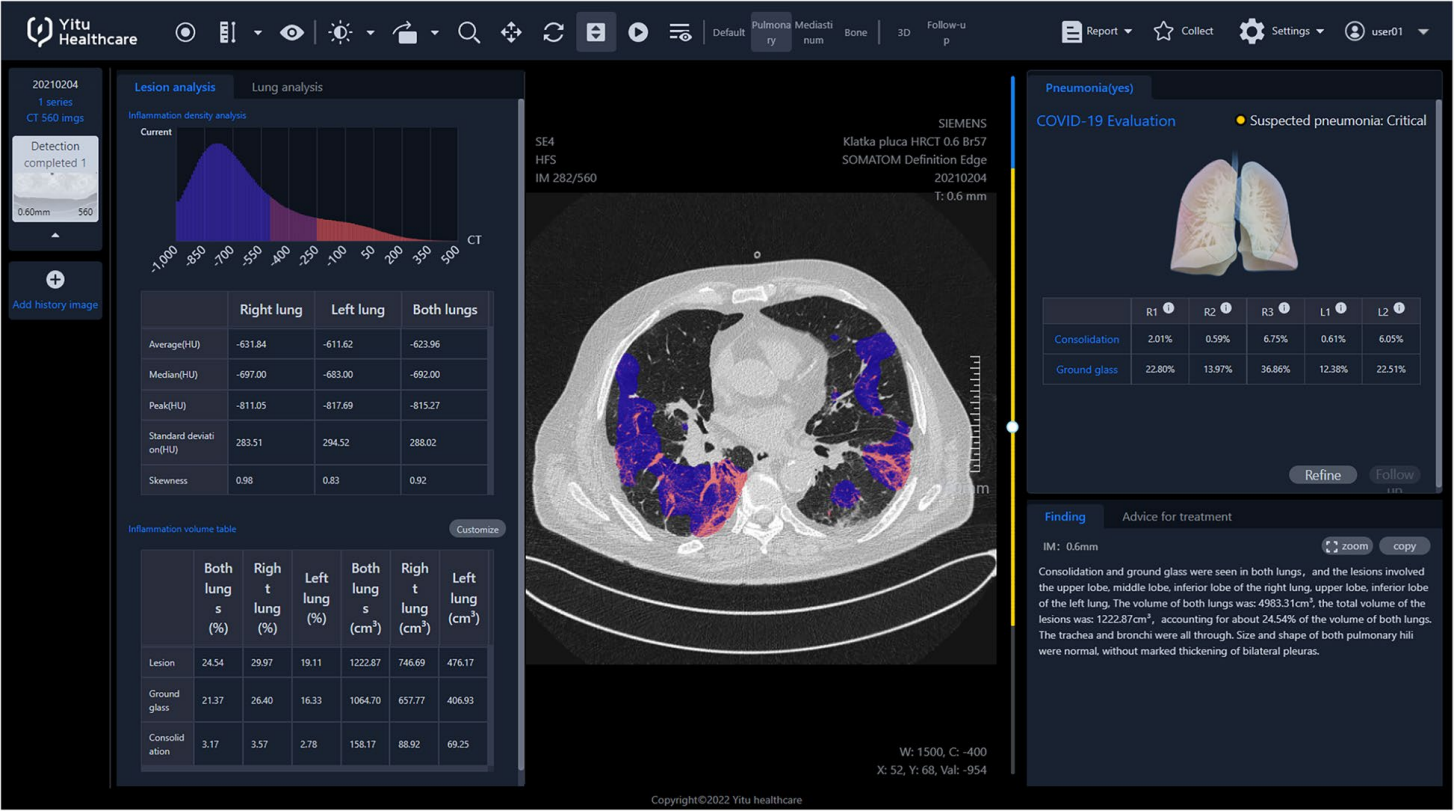
The final report from automatic analysis of HRCT by artificial intelligence technology

The analysis of the extent of pulmonary lesions in HRCT images was performed by AI software created by YITU CT, YITU Healthcare Technology Co., Ltd. in cooperation with Huawei Technologies Co., Ltd., China [18, 19] (Fig. 2).

The structure of the YITU CT AI software is described in the publication by Pan et al. [20]. The system includes three network parts: (1) twelve convolutional segments (convolutional, batch normalization, and activation layers); (2) tree max-pooling layers performing down-sampling; and (3) tree transpose convolutional layers performing up-sampling. The chest CT images from 942 COVID-19 patients and 1340 healthy persons, were used to train the system. According to the authors, the Dice coefficient determining the accuracy of the lung inflammation volume measurement by AI, in reference to the ground truth volume measurement by experienced radiologists, was 85.00% for the training set and 82.08% for the test set.

The YITU CT is a commercially available product, and it was purchased by the Krakow University Hospital to use during the CRACoV-HHS project.

For the purposes of this study, the software automatically calculated the percentage inflammation volume (PIV) in relation to the whole lung tissue volume for: both lungs, right lung, left lung, right upper lobe (RUL), right middle lobe (RML), right lower lobe (RLL), left upper lobe (LUL), and left lower lobe (LLL). Additionally, AI divided the whole inflammation volume into ground glass and consolidation volumes; therefore the software also provided the percentage ground glass volume (PGV), and the percentage consolidation volume (PCV) in relation to the whole lung tissue volume, consecutively as above for: both lungs, right lung, left lung, RUL, RML, RLL, LUL, and LLL. Typically, in the initial phase of COVID-19 pneumonia, ground glass dominates in the whole inflammation volume, and only later consolidation volume increases. The value of PCV divided by PGV reflects the relation of consolidation to ground glass, in the whole volume of inflammation.

Besides, the system calculated CT score values, representing the degree of lung tissue involvement by inflammatory changes – every lobe was scored 0 – 10, thus right lung 0 – 30, left lung 0 – 20, both lungs 0 – 50. Finally, AI reported the estimated degree of pneumonia severity, based on CT evaluation, defined as none, mild, moderate, or critical.

### Inflammatory markers at admission

Laboratory markers of inflammation analyzed included: CRP (C-reactive protein), IL6 (interleukin 6) and PCT (procalcitonin), and were obtained within 24 h from LUS.

### Clinical assessment of respiratory function throughout hospitalization

3 subgroups of patients were distinguished depending on the respiratory failure throughout the hospitalization period:

- respiratory efficient – not requiring oxygen therapy,
- requiring low-flow oxygen therapy with FiO_2_ (fraction of inspired oxygen) < 50%,
- requiring advanced respiratory support, including: oxygen therapy with FiO_2_ ≥ 50%, HFNOT (high flow nasal oxygen therapy), CPAP (continuous positive airway pressure) or invasive ventilation.

### Statistical analysis

First, we tested the correlations between the inflammation volume assessed in LUS and in HRCT. We analyzed the correlations between LUS score and CT score, as well as between LUS score and PIV, consecutively for both lungs, right lung, left lung, RUL, RML, RLL, LUL, LLL.

Then we tried to assess the correlations between LUS results and the structure of inflammation in HRCT. We analyzed the correlations between LUS score and the value of PCV divided by PGV, consecutively for both lungs, right lung, left lung, RUL, RML, RLL, LUL, LLL.

In the next step we analyzed the correlations between LUS score for both lungs and the laboratory markers of inflammation: CRP, IL6, PCT, and similarly between CT score for both lungs and CRP, IL6, PCT.

Finally, we calculated the values of median LUS score for both lungs in three subgroups of respiratory function: not requiring oxygen therapy, requiring low-flow oxygen therapy with FiO_2_ < 50%, and requiring advanced respiratory support. We assessed the statistical significance of differences between the LUS scores in the above three subgroups.

Due to non-normal distributions, the correlations were evaluated using the Spearman correlation coefficient. The comparison of the values in the subgroups was performed using the Kruskal–Wallis test, and after detecting statistically significant differences, post-hoc analysis with Dunn’s test was performed. The *p*-value < 0.05 was considered statistically significant. Statistical analyses were performed using Statistica 13.3 software (TIBCO Software Inc., Palo Alto, CA, USA).

## Results

### Study population and timing of imaging

This study comprised 65 patients: 27 women, 38 men, 23–87 years old, average, and median 63 years old. HRCT was performed on the same day as LUS in 16 patients (24.6%). In others the interval accounted to 1, 2 and 3 days for 32 (49.2%), 14 (21.5%) and 3 (4.6%) patients, respectively.

### Results of imaging

For both lungs, the median LUS score was 19 (IQR—interquartile range 11–24) and the median HRCT score was 22 (IQR 16–26).

In LUS bilateral lung involvement was observed in 58 (89.2%) patients and more than one lobe was involved in 59 (90.1%) patients.

In HRCT bilateral lung involvement was present in 61 (93.8%) patients and more than one lobe was involved in 63 (92.6%) patients.

PGV ranged from 0.03 to 60.14%, and the number of patients with PGV < 1.0% was 6 (9.2%).

PCV ranged from 0.01 to 18.82%, and the number of patients with PCV < 1.0% was 25 (38.5%).

The severity of pneumonia was assessed by AI in HRCT as critical in 37 (56.9%) cases, moderate in 17 (26.2%) cases, mild in 10 (15.4%) cases, none in 1 patient.

Detailed results of LUS and HRCT stratified by the lung regions are presented in Table 2.

**Table 2:** LUS and HRCT results stratified by the lung regions

Lung region	LUS score	Maximal available LUS score	CT score	Maximal available CT score	PIV, %	PGV, %	PCV, %
Both lungs	19 (11–24)	48	22 (16–26)	50	12.5 (5.0–21.8)	9.1 (4.6–17.2)	1.9 (0.6–4.0)
Right lung	10 (4–12)	24	12 (9–15)	30	14.9 (3.5–24.4)	11.9 (3.3–17.5)	2.1 (0.5–4.6)
Left lung	10 (5–13)	24	7 (5–10)	20	8.6 (3.6–21.4)	8.1 (3.4–17.7)	1.4 (0.3–3.8)
RUL	5 (2–7)	12	5 (2–5)	10	8.4 (1.8–26.1)	7.4 (1.5–21.1)	0.9 (0.2–3.5)
RML	1 (0–2)	3	2 (2–5)	10	6.7 (1.0–15.6)	6.4 (0.9–14.3)	0.4 (0.1–1.4)
RLL	3 (2–5)	9	5 (5–7)	10	15.5 (6.4–35.7)	14.0 (5.2–25.3)	2.9 (0.8–8.5)
LUL	6 (3–8)	15	5 (2–5)	10	6.0 (1.4–19.2)	5.5 (1.2–15.1)	0.5 (0.1–1.7)
LLL	4 (2–6)	9	5 (2–5)	10	13.4 (4.4–32.1)	10.9 (3.2–22.8)	2.2 (0.4–5.7)

### Correlations between the inflammation volume assessed in LUS and in HRCT

The analysis revealed significant correlations between LUS and CT scores in all evaluated lung regions. The strength of correlation was the highest for right lung (*r* = 0.76) and both lungs (*r* = 0.75). The detailed results are presented in Table 3.

**Table 3:** The correlations between LUS and CT scores stratified by the lung regions

**Location**	**Spearman Rank Order Correlations** All correlations are significant at *p* < 0.05
Both lungs	0.75
Right lung	0.76
Left lung	0.64
RUL	0.68
RML	0.65
RLL	0.52
LUL	0.61
LLL	0.62

The correlations between LUS score and PIV were slightly weaker but remained statistically significant. The strongest correlations were observed for right lung (*r* = 0.70), both lungs (*r* = 0.69), left lung (*r* = 0.68), and RUL (*r* = 0.68). Detailed results are summarized in Table 4.

**Table 4:** The correlations between LUS score and PIV stratified by the lung regions

**Location**	**Spearman Rank Order Correlations** All correlations are significant at *p* < 0.05
Both lungs	0.69
Right lung	0.70
Left lung	0.68
RUL	0.68
RML	0.62
RLL	0.56
LUL	0.67
LLL	0.59

### The correlations between LUS results and the structure of inflammation

The correlations between LUS score and the value of PCV divided by PGV, were weak or not significant. Detailed results are summarized in Table 5.

**Table 5:** The correlations between LUS score and the value of PCV divided by PGV, stratified by the lung regions

**Location**	**Spearman Rank Order Correlations** Significant correlations at *p* < 0.05 are marked by *
Both lungs	0.24
Right lung	0.14
Left lung	0.34*
RUL	0.20
RML	0.33*
RLL	0.24
LUL	0.48*
LLL	0.21

### The correlations between LUS score for both lungs and the laboratory markers of inflammation

We found significant correlation between LUS score for both lungs and CRP (*r* = 0.55, *p* < 0.001), as well as between LUS score for both lungs and IL6 (*r* = 0.39, *p* = 0.003) but not between LUS score for both lungs and PCT (*r* = 0.15, *p* = 0.23). Similarly, CT score for both lungs was correlated with CRP (*r* = 0.70, *p* < 0.001) and IL6 (*r* = 0.54, *p* < 0.001) but not with PCT (*r* = 0.22, *p* = 0.084).

### Differences between the LUS scores in the respiratory function subgroups

There were 22 patients not requiring oxygen therapy, 33 patients requiring low-flow oxygen therapy with FiO_2_ < 50%, and 10 patients requiring advanced respiratory support throughout the hospitalization.

The median values of initial LUS score for both lungs in the above subgroups were respectively: 9.0, 22.0, 30.5. Post-hoc tests revealed statistically significant differences between: advanced respiratory support group and low-flow oxygen therapy group (*p* = 0.01), advanced respiratory support group and the no oxygen group (*p* < 0.001), as well as low-flow oxygen group and no oxygen group (*p* < 0.001).

## Discussion

This prospective observational study on patients hospitalized due to COVID-19 confirmed a strong correlation between the extent of inflammation found in LUS and in HRCT.

Significant correlations were also found between the degree of lung involvement in LUS and laboratory markers of inflammation at admission.

Finally, we found an association between initial LUS scores and the degree of respiratory failure throughout the hospitalization.

This study suggests that LUS can be regarded as an accurate method to evaluate the extent of COVID-19 pneumonia and could be considered as a potentially promising tool to estimate its clinical severity.

Our data is consistent with the results of other authors.

Tung-Chen et al. in a group of 51 COVID-19 patients admitted to emergency department, found a strong correlation (*r* = 0.803) between LUS score and CT total severity score assessed by radiologists. [21].

Similarly, Tana et al. in a larger group of 153 COVID-19 patients reported a strong (r = 0.754) correlation between LUS score and chest CT score [22].

Nouvenne et al. in a group of 26 patients, urgently hospitalized for COVID-19 pneumonia, found that LUS score was significantly correlated with CT visual scoring (*r* = 0.65, *p* < 0.001) and oxygen saturation in room air (*r* = –0.66, *p* < 0.001) [23].

Also other previous studies reported that diagnostic agreement between LUS and CT in the assessment of COVID-19 pneumonia is high, however in majority of them CT was not performed using a dedicated HRCT protocol for precise pulmonary tissue assessment [24].

Evidence accumulated through two years of pandemic showed good sensitivity of LUS in detecting COVID-19 pneumonia but poor specificity, which was significantly lower in comparison to HRCT [2].

Allinovi et al. in a review article concluded that LUS may be a first-line diagnostic imaging alternative to chest CT and X-ray during every step of COVID-19 disease, even before clinical manifestations, particularly in children, pregnant women, critically ill patients, and patients in areas with high rates of community transmission [25].

Nouvenne et al. performed LUS in 83 older nursing home residents presenting mild to moderate respiratory symptoms and not previously tested for COVID-19. The conclusion was that LUS may represent a valid diagnostic aid for an early detection of COVID-19 outbreaks and adequate patient management [26].

In the presented study both LUS and HRCT confirmed that in vast majority of patients with COVID-19 there is a bilateral lung involvement. The percentages of patients with bilateral lung involvement and with more than one lobe involvement were slightly lower on LUS compared to HRCT.

The main innovation in our study was the application of AI for automatic and objective assessment of HRCT images. In effect, the analysis of the extent of lung involvement in HRCT was not a time-consuming manual task using dedicated scoring systems, but it was performed automatically with reports available within minutes.

Using AI it was possible not only to compare LUS and HRCT extent of inflammation for both lungs, as in the research of other authors, but also separately for right, left lung, and individual lobes.

Our analysis showed that correlation of LUS and HRCT becomes weaker when individual lobes are evaluated. This is probably related to only approximate assignments of chest area segments used during LUS to the anatomical locations of lung lobes and variable number of LUS segments for each lobe (from 1 for RML, up to 5 for LUL). Therefore, we believe that LUS should be considered as a tool for global evaluation of lung involvement, while HRCT should remain a standard when precise evaluation of pulmonary lesions is necessary.

Hospitalized patients with COVID-19 require regular reevaluation of lung involvement. Therefore, LUS is considered a very promising alternative to HRCT as it allows to monitor the disease dynamics without exposing patients to excessive ionizing radiation. Several previous reports additionally showed evidence that both LUS score and CT score may be prognostic factors in patients with COVID-19 [15, 27–29].

In our study, despite the exclusion of patients with SpO^2^ < 94% at admission, the severity of pneumonia was assessed by AI in HRCT as critical in 37 (56.9%) of cases.

In our previous article [15] concerning 804 patients with COVID-19 pneumonia and HRCT analyzed by the same AI, in the subgroup of 480 patients reported as “critical”, the median value of PIV was 30.64 (17.96–46.41) %, and the median value of SpO^2^ at admission was 93 (89–96) %.

It means, that the term “critical” used by the AI software creators should be rather regarded as “severe” (inflammation occupying about one third of the whole lung volume), and even in this group many patients may have SpO^2^ at admission ≥ 94%.

The secondary aim of our study was to evaluate whether LUS is useful in the assessment of the structure of inflammation, by differentiation of ground glass from consolidations.

In a theoretical model two different phenotypes of pulmonary lesions in COVID-19 pneumonia have been described: the more frequent ‘L’ phenotype, in which HRCT typically presents peripheral ground glass areas, and the less frequent ‘H’ phenotype with dense lobar consolidations in HRCT [30]. Some authors believe that LUS is potentially able to differentiate these two phenotypes, based on the different patterns [31]. Especially, LUS score 3 used for large consolidations should be typical for ‘H’ phenotype.

In our study the correlations between LUS score and the value of PCV divided by PGV, turned out to be weak or not significant.

The reason may be the fact that most of the patients in our study were at the initial stage of the COVID-19 pneumonia, with dominant ground glass lesions. LUS score equal to 3 was given in less than 1% of all the examined chest areas. Therefore the analysis of the value of LUS in the assessment of the structure of inflammation, requires further studies in the groups of patients during monitoring the course of the disease.

Our results are in line with other studies reporting association of LUS findings with clinical severity of Covid-19 infection including respiratory impairment and the level of inflammatory markers [32–35]. While previous data showed reliability of LUS in predicting the need of hospitalization or intensive care or intubation, our results indicate that LUS score on admission may be related to the intensity of further respiratory support, thus the predictive value of LUS may be even more precise than previously thought.

We are aware of several limitations of our study. The study sample was relatively low which limits the power of the results. Some analyses were made on the basis of assumptions concerning e.g. relation of probe placement and corresponding lung lobe, which definitely affects the results. Addition of extended clinical data (demographic, comorbidities, vital parameters, treatment, outcomes) would definitely enrich this study, e.g. for example, enabling the assessment of the prognostic factors of in-hospital death.

In addition, COVID-19 is a dynamic disease and the interval between LUS and HRCT could impact the degree of agreement between results of these examinations, however this interval did not exceed 1 day in approximately 75% of study sample.

B-lines in LUS are not specific for COVID-19 pneumonia but are also found in fibrotic-type interstitial changes. The presented study does not explain whether LUS is useful in differentiation between different patterns of interstitial changes (ground glass vs. reticular changes vs. honeycombing), because AI software did not categorize interstitial involvement into these subgroups and we did not collect precise data on regularity and thickness of pleural line.

In LUS it is difficult to evaluate interstitial involvement if it is not subpleural. This limitation of LUS may have also influenced the level of correlation with HRCT, despite the fact that COVID-19 pulmonary changes are predominantly peripheral.

In our study we used HRCT in all the patients with inflammatory changes found in LUS. As already stated, the guidelines do not recommend HRCT as a screening tool, nor as a first-line test, but suggest performing it in complicated COVID-19 cases. Our goal was to check whether and to what extent LUS can replace HRCT in COVID-19 in the future. That is why the Ethical Board accepted using HRCT in our study as a gold standard for LUS assessment.

The last limitation concerns the lack of inclusion of patients with respiratory insufficiency, which makes it impossible to determine to what degree the correlation between LUS and HRCT results applies to the most severe forms of the disease.

In conclusion, LUS can be regarded as an accurate method to evaluate the extent of COVID-19 pneumonia and as a promising tool to estimate its clinical severity. Evaluation of LUS in the assessment of the structure of inflammation, requires further studies in the course of the disease.

## Data Availability

The data that support the findings of this study are available from the CRACoV-HHS project at the Krakow University Hospital, but restrictions apply to the availability of these data, which were used under license for the current study, and so are not publicly available. Data are however available from the authors upon reasonable request and with permission of the CRACoV-HHS project management. The corresponding author is the contact person for data availability requests.

## Abbreviations

AI: Artificial intelligence
CPAP: Continuous positive airway pressure
CRP: C-reactive protein
FiO_2_: Fraction of inspired oxygen
HFNOT: High flow nasal oxygen therapy
HRCT: High resolution computed tomography
IL6: Interleukin 6
LLL: Left lower lobe
LUL: Left upper lobe
LUS: Lung ultrasound
PCR: Polymerase chain reaction
PCT: Procalcitonin
PCV: Percentage consolidation volume
PGV: Percentage ground glass volume
PIV: Percentage inflammation volume
RLL: Right lower lobe
RML: Right middle lobe
RUL: Right upper lobe
